# Outbreak of Multidrug Resistant NDM-1-Producing *Klebsiella pneumoniae* from a Neonatal Unit in Shandong Province, China

**DOI:** 10.1371/journal.pone.0119571

**Published:** 2015-03-23

**Authors:** Yan Jin, Chunhong Shao, Jian Li, Hui Fan, Yuanyuan Bai, Yong Wang

**Affiliations:** 1 Department of Microbiology, Clinical Laboratory of Provincial Hospital affiliated to Shandong University, Jinan, PR China; 2 Jinan Centre for Disease Control and Prevention, Jinan, PR China; Iowa State University, UNITED STATES

## Abstract

Despite worldwide dissemination of New Delhi metallo-β-lactamase1 (*bla*NDM-1), outbreaks remain uncommon in China. In this study, we describe the characteristics of the outbreak-related *bla*NDM-1-producing *K*. *pneumonia* isolates in a neonatal unit in Shandong province, China. We recovered 21 non-repetitive carbapenem-resistant *K*. *pneumoniae* isolates with a positively modified Hodge test (MHT) or EDTA synergistic test from patients and environmental samples in Shandong provincial hospital. Pulsed-field gel electrophoresis (PFGE) and multilocus sequence typing (MLST) data show *K*. *pneumoniae* isolates from 19 patients were clonally related and belong to the clonal groups ST20 and ST17. We note two outbreaks, the first caused by ST20 during August 2012 involving four patients, and the second caused by ST20 and ST17 during January 2012 and September 2013 involving fourteen patients. We found the bed railing of one patient was the source of the outbreak. We verified the presence of the *bla*NDM-1 gene in 21 *K*. *pneumoniae* isolates. The genes *bla*CTX-M-15, *bla*CTX-M-14, *bla*DHA-1, *bla*TEM-1 and Class I integron were also present in 18 (85.7%), 3 (14.3%), 18 (85.7%), 19 (90.5%) and 19 (90.5%) isolates, respectively. We also found an isolate with both *bla*NDM-1 and *bla*IMP-4. All of the isolates exhibited a multidrug-resistance phenotype. The β-lactam resistance of 20 isolates was transferable via conjugation. In addition, we show the resistance of 21 *K*. *pneumoniae* isolates to carbapenem is not related to lack of outer-membrane proteins OmpK35 and OmpK36 nor overpression of efflux pumps. This study provides the first report confirming blaNDM-1-producing *K*. *pneumoniae* ST20 and ST17 were associated with outbreak. Early detection of resistance genes is an effective strategy in preventing and controlling infection by limiting the dissemination of these organisms.

## Introduction

*K*. *pneumonia* is a main pathogen in communities and hospitals. It causes infection in the respiratory tract, urinary tract, bloodstream and abdominal cavity. Carbapenem is the most effective antibiotic for treatment of gram negative bacilli that are resistant to β-Lactamases. However, in recent years, widespread use of carbapenem has accelerated the growth of carbapenem-resistant strains with reports in many countries [1–3].

Carbapenem resistance in enterobacteriaceae is mainly caused by two mechanisms: one by the production of carbapenemase (including class A serine carbapenemase: *bla*KPC, *bla*GES, *bla*SME, *bla*NMC, *bla*IMI; class B metal enzyme: *bla*NDM, *bla*IMP, *bla*VIM, *bla*SIM, *bla*SPM, *bla*GIM; class D enzyme: *bla*OXA); and the second by an absence or decreased expression of outer membrane proteins. In addition, mutations in the AMPC enzyme or extended-spectrum-β-lactamases (ESBL) play an important role in the drug-resistance mechanisms.

Class A and class B carbapenemases, especially *bla*KPC, *bla*IMP and *bla*NDM, are more commonly reported in enterobacteriaceae. Steinmann *et al*. reported on the hospital outbreak caused by *bla*KPC-2-producing *K*. *pneumoniae* in July 2010 - January 2011 in Germany that resulted in four deaths[4]. In March 2011, *bla*NDM-1-producing *E*.*coli* was isolated in Hong Kong, China [5]. Occurrences of carbapenem-resistant enterobacteriaceae (CRE) have been rising gradually in recent years in Mainland China, and have caused dissemination of nosocomial infection. An outbreak of 77 *bla*KPC-2-producing enterobacteriaceae was reported in Shanghai Huashan hospital in 2012, severely stressing the ability of hospitals and clinics to control the infection[6]. Fortunately, an outbreak of *bla*NDM-1-producing *K*.*pneumoniae* has not been reported in Chinese hospitals.

In this study, we collected 21 carbapenem-resistant *K*. *pneumoniae* from patients in the neonatal and cardiac surgery ward from August 2012 to August 2013. We investigated the drug-resistance and transmission mechanisms of carbapenem-resistant strains and homogeneity of strains.

## Materials and Methods

### Collection of isolates

Twenty-one non-duplicated carbapenem-resistant *K*. *pneumoniae* were included in this study. Twenty isolates were from neonatal and one from cardiac surgery during August 2012 to September 2013 in Shandong province, China. Swabs were collected from hands and noses of doctors and nursing staff in the neonatal unit; and environmental cultures were collected using a swab pre-moistened with sterile saline to identify other potential reservoirs for blaNDM-1 producing *K*. *pneumoniae*. Cultured sites included bed railing, stethoscope, weighing machine, milk powder, suction apparatus, wash basin, radiant warmer, washing area, electric switch, medicine tray and ventilator machine. All isolates were identified by a VITEK-2 compact system and screened for carbapenemase production by the modified Hodge test (MHT) and ertapenem-EDTA double-disc synergistic test [7]. Informed consent was obtained from the participants before the study. The participants provide written informed consent to participate in this study. The subjects’ rights and interests were protected well in the research. This study and consent procedure was approved by the Medical Ethics Committee of Shandong Provincial Hospital affiliated with Shandong University.

### Susceptibility testing

Antibiotic susceptibility testing was assayed by the agar dilution method. We measured minimum inhibitory concentration (MICs) of cefotaxime, ceftazidime, cefepime, cefoxitin, piperacillin-tazobactam, trimethoprim-sulfamethoxazole, meropenem, imipenem, ertapenem, gentamicin, levofloxacin, amikacin, aztreonam, tigecycline, colisin and fosfomycin. All antibiotics, except tigecycline and colisin, were interpreted according to the approved standard of Clinical and Laboratory Standards Institute (CLSI) 2013 guidelines [8]. The 2013 European Committee on Antimicrobial Susceptibility Testing breakpoint was used (available at http://www.eucast.org/clinicalbreakpoint) for colistin and tigectcline. *E*.*coli ATCC25922* was used for quality control.

### PCR and DNA sequence analysis of drug-resistance genes

We screened carbapenem-resistance genes (*bla*KPC, *bla*SME, *bla*IMI/*bla*NMC, *bla*GES, *bla*IMP, *bla*VIM, *bla*GIM, *bla*SIM-1, *bla*SPM, *bla*NDM-1 and *bla*OXA), common ESBL genes (*bla*CTX-M, *bla*TEM, and *bla*SHV), AMPC genes (*bla*MOX, *bla*FOX, *bla*DHA, *bla*CIT, and *bla*EBC), as well as integron structures (Int1, Int2.Int3) in all clinical strains using previously described primers [9–12]. The positive PCR products were screened by electrophoresis on 1.0% agarose gel and sequenced by Shanghai Majorbio Bio-Pharm Technology Co. (Shanghai, China). Nucleotide sequences were analyzed and compared using BLAST (http://www.ncbi.nlm.nih.gov/BLAST).

### Resistance gene transfer experiments

Conjugation experiments were performed using azide-resistant *E*.*coli* J53 as the recipient strain. Briefly, overnight cultures of the donor strain (500 ml) and recipient strain (500 ml) were mixed with 10 ml fresh Mueller-Hinton broth and incubated for 24 h at 35°C. Mixture was inoculated on MacConkey agar plates containing sodium azide (200 ug/ml) and imipenem (0.5 ug/ml) for 24 h at 35°C. Conjugation was confirmed by indole testing. Presence of carbapenemase gene was confirmed by PCR analysis.

### Plasmid profiling

Plasmid DNA of transconjugants was purified using a QuickGene plasmid kit S (Fuji, Tokyo, Japan) according to the manufacturer's instructions, followed by electrophoresis in a 0.7% agarose gel at 90 V for 3 h [13]. The sizes of the plasmids were compared by co-electrophoresis with plasmids of known sizes from *E*. *coli* (V517and 39R861). Bands were visualized with UV transilluminator after staining.

### Inhibitory effects of CCCP against efflux

We determined presence of efflux pump in carbapenem-resistant enterobacteriaceae by CCCP inhibition test. MIC changes were observed in the absence or presence of CCCP (Sigma) at concentrations of 50μg/mL. An inoculum of each isolate (5 × 10^4^ cfu/mL) was inoculated onto MH medium containing serial dilutions of imipenem and meropenem. A phenotype for positive efflux was detected after at least 4-fold dilutions of MIC for imipenem or meropenem in the absence or presence of CCCP [14].

### Pulse-field gel electrophoresis (PFGE)

An overnight-grown bacterial culture was suspended in EET buffer (100 mM EDTA, 10 mM EGTA, 10 mM Tris—HCl (pH 8)) and adjusted to an optical density of 0.9 at 600 nm. The suspension was mixed with equal volumes in a 2% solution of low-melting-temperature agarose in EET buffer. After solidification, the agarose plugs were incubated for 4 h at 37°C in EET buffer containing 1 mg lysozyme and 50 ug lysostaphin per ml. The plugs were transferred to EET buffer containing 1% sodium dodecy1 sulfate and 20 mg proteinase K per ml of buffer and incubated overnight at 50°C. Plugs were washed thoroughly with TE buffer (10mM Tris—HCl, 1 mM EDTA (pH 8)) and digested overnight with XbaI restriction endonuclease (TAKARA, Shiga, Japan). DNA separation was performed in 0.5× TBE buffer in a pulsed-field electrophoresis system (CHEF MAPPER; Bio-Rad Laboratories, California, USA) with the following conditions: temperature 14°C; voltage 6 V/cm; switch angle, 120°and switch ramp of 4–40 s for 21h. *Salmonella enterica serotype Braenderup* H9812 was used as a marker for PFGE. The restriction patterns were analyzed and interpreted according to Tenover et al [15].

### Multilocus sequence typing (MLST)

MLST of *K*.*pneumoniae* was performed using seven conserved housekeeping genes (*bla*gapA, *bla*infB, *bla*mdh, *bla*pgi, *bla*phoE, *bla*rpoB and *bla*tonB) according to protocols available at the MLST Pasteur website (http://www.pasteur.fr/recherche/genopole/PF8/mlst/Kpneumoniae.html).

### Analysis of outer membrane proteins (OMPs)

The genes of *bla*OMPs were screened in all clinical isolates using previously described primers by PCR [16]. PCR negative isolates were investigated for alterations in the OMPs by Sodium dodecy1 sulfate-polyacrylamide gel electrophoresis (SDS-PAGE) as described previously [17].

## Results

### Clinical characteristics of 21 *K*. *pneumoniae*

Table 1 shows the clinical characteristics of the 21 carbapenem-resistant *K*. *pneumoniae*, including isolation date, specimen and ward distribution. The first strain was obtained on August 1, 2012, with three additional samples isolated from the neonatal unit. No additional isolates were observed between September and December. However, beginning in January 2013, the isolation rate significantly increased in the same ward, with 15 strains isolated from January to August. On September 10, 2013, one strain was isolated from a cardiac surgery patient with congenital heart disease. We isolated one from the bed railing of patient (H16) in September as well. Cultures from physicians, nurses and environmental samples were negative.

**Table 1:** Characteristics of 21 *K*. *pneumoniae* isolates with NDM-1.

Isolate No.	Sex	Patient age	Ward	Isolate date	Specimen	HTM	EDTA	PFGE pattern	Sequence type	Resistance gene			Class I integrons
										Carbapenemase	ESBL	AMPC	
H1	M	30d	neonatal unit	2012/8/1	sputum	-	+	cluster1	20	blaNDM-1	blaTEM-1,blaCTX-M-15	blaDHA-1	+
H2	M	21d	neonatal unit	2012/8/23	sputum	-	+	cluster1	20	blaNDM-1	blaTEM-1,blaCTX-M-15	blaDHA-1	+
H3	M	1d	neonatal unit	2012/8/29	sputum	±	-	cluster1	20	blaNDM-1	blaTEM-1,blaCTX-M-15	blaDHA-1	+
H4	M	13d	neonatal unit	2012/8/30	sputum	-	+	cluster1	20	blaNDM-1	blaTEM-1,blaCTX-M-15	blaDHA-1	+
H5	M	60d	neonatal unit	2013/1/1	sputum	-	+	cluster1	20	blaNDM-1	blaTEM-1,blaCTX-M-15	blaDHA-1	+
H6	F	22d	neonatal unit	2013/1/29	sputum	-	+	cluster1	20	blaNDM-1	blaTEM-1,blaCTX-M-15	blaDHA-1	+
H7	M	30d	neonatal unit	2013/2/25	sputum	-	+	cluster1	20	blaNDM-1	blaTEM-1,blaCTX-M-15	blaDHA-1	+
H8	M	56d	neonatal unit	2013/3/5	sputum	±	+	cluster1	20	blaNDM-1	blaCTX-M-15	blaDHA-1	+
H9	M	28d	neonatal unit	2013/3/6	sputum	-	+	cluster1	20	blaNDM-1	blaTEM-1,blaCTX-M-15	blaDHA-1	+
H10	F	25d	neonatal unit	2013/3/14	sputum	-	+	cluster1	20	blaNDM-1	blaTEM-1,blaCTX-M-15	blaDHA-1	+
H11	M	32d	neonatal unit	2013/3/19	sputum	-	+	cluster1	20	blaNDM-1	blaCTX-M-15	blaDHA-1	+
H12	M	34d	neonatal unit	2013/4/19	sputum	-	-	cluster1	20	blaNDM-1	blaTEM-1,blaCTX-M-15	blaDHA-1	+
H13	M	13d	neonatal unit	2013/4/23	umbilicus	+	-	cluster4	54	blaNDM-1	blaTEM-1,blaCTX-M-15	blaDHA-1	+
H14	F	15d	neonatal unit	2013/7/10	sputum	-	-	cluster3	20	blaNDM-1	blaTEM-1,blaCTX-M-15	blaDHA-1	+
H15	M	9d	neonatal unit	2013/7/13	sputum	-	-	cluster1	20	blaNDM-1	blaTEM-1,blaCTX-M-15	blaDHA-1	+
H16	M	35d	neonatal unit	2013/7/21	sputum	+	-	cluster1	20	blaNDM-1	blaTEM-1,blaCTX-M-15	blaDHA-1	+
J17			neonatal unit	2013/7/24	bed railing	-	+	cluster1	20	blaNDM-1	blaTEM-1,blaCTX-M-15	blaDHA-1	+
H18	M	1d	neonatal unit	2013/7/27	sputum	+	+	cluster2	17	blaNDM-1	blaTEM-1,blaCTX-M-14	-	-
H19	F	26d	neonatal unit	2013/7/29	blood	±	+	cluster1	20	blaNDM-1	blaTEM-1,blaCTX-M-14	blaDHA-1	+
H20	F	2d	neonatal unit	2013/8/19	sputum	-	+	cluster2	17	blaNDM-1	blaTEM-1,blaCTX-M-15	-	-
H21	F	1y	cardiac surgery	2013/9/10	sputum	±	+	cluster2	17	blaNDM-1,blaIMP-4	blaTEM-1,blaCTX-M-14	-	dfrA1-orfC

Kpn: *Klebsiella pneumoniae*, M: male, F: female, EDTA: EDTA synergistic test, HTM: modified Hodge test, ESBL: extended-spectrum-β-lactam

19 isolates were from newborns in the neonatal unit, including 14 males and 5 females. Eighteen newborns had pneumonia and one had umbilical infection. All were premature with low birth weight. Nine had intrauterine infection at birth. All were previously treated with various antimicrobial agents including cloxacillin, cefathiamidine and ceftizoxime. Eight were treated with imipenem or meropenem. The patient from cardiac surgery had respiratory tract infection and fever five days after operation.

### Susceptibility results

The MICs of each antibiotic are shown in Table 2. 21 *K*. *pneumoniae* isolates carrying *bla*NDM-1 exhibited resistance to cefotaxime, ceftazidime, cefepime, cefoxitin, piperacillin-tazobactam, meropenem, imipenem and ertapenem. Tigecycline, colistin, levofloxacin and amikacin showed strong activity against carbapenem-resistant *K*. *pneumoniae* with a susceptibility rate of 100%. The antibiotic resistant rates of gentamicin, aztreonam, trimethoprim-sulfamethoxazole and fosfomycin were 14.28% (3/21), 95.24% (20/21), 23.81% (5/21), and 4.76% (1/21), respectively. It is worth noting that based on MLST results, these gentamicin-resistant *K*. *pneumoniae* all belong to ST17, of which two were derived from neonatal unit and one from cardiac surgery.

**Table 2:** The antibiotic susceptibility of blaNDM-producing *k*. *pneumonia* (μg/ml).

isolate No.	IMP	MEM	ETP	CN	AK	TZP	CTX	CAZ	FEP	FOX	LEV	SXT	TGC	CO	FOS	ATM
H1	8	8	>32	0.5	2	>256	>256	>256	>256	>256	0.5	>32	0.5	0.125	32	>256
H2	24	8	>32	0.5	4	>256	>256	>256	>256	>256	0.5	0.125	0.5	0.125	48	>256
H3	>32	>32	>32	0.5	2	>256	>256	>256	>256	>256	1	0.125	0.5	0.125	48	>256
H4	>32	>32	>32	1	4	>256	>256	>256	>256	>256	1	0.125	0.5	0.125	32	>256
H5	>32	>32	>32	1	4	>256	>256	>256	>256	>256	0.5	0.25	0.5	0.125	16	>256
H6	>32	>32	>32	1	2	>256	>256	>256	>256	>256	1	0.125	0.5	0.125	32	>256
H7	>32	>32	>32	0.5	2	>256	>256	>256	>256	>256	1	0.25	0.5	0.125	16	>256
H8	>32	>32	>32	0.5	2	>256	>256	>256	>256	>256	1	0.25	0.25	0.125	48	>256
H9	32	32	32	0.5	4	>256	>256	>256	>256	>256	1	0.25	1	0.125	24	>256
H10	>32	>32	>32	0.5	2	>256	>256	>256	>256	>256	1	0.25	0.5	0.032	48	>256
H11	>32	>32	>32	0.5	2	>256	>256	>256	>256	>256	1	0.25	0.5	0.032	32	>256
H12	>32	>32	>32	1	2	>256	>256	>256	>256	>256	1	0.125	0.5	0.125	48	>256
H13	4	32	32	0.5	4	>256	>256	>256	>256	>256	1	>256	1	0.125	>256	1
H14	32	32	>32	0.5	2	>256	>256	>256	>256	>256	1	0.125	1	0.25	48	>256
H15	>32	>32	>32	0.5	4	>256	>256	>256	>256	>256	1	0.125	0.5	0.25	48	>256
H16	>32	>32	>32	0.5	4	>256	>256	>256	>256	>256	0.5	0.5	1	0.25	16	>256
J17	>32	>32	>32	0.5	4	>256	>256	>256	>256	>256	0.5	0.5	0.5	0.25	32	>256
H18	>32	8	>32	32	2	>256	>256	>256	>256	>256	0.5	>32	1	0.25	16	>256
H19	>32	>32	>32	0.5	4	>256	>256	>256	>256	>256	0.5	0.5	1	0.25	48	>256
H20	>32	4	32	32	2	>256	>256	>256	>256	>256	1	>32	0.5	0.125	16	>256
H21	>32	>32	>32	16	2	>256	>256	>256	>256	>256	0.5	>32	1	0.125	16	>256

MIC: Minimum inhibitory concentration, CTX: cefotaxime, CAZ: ceftazidime, FEP: cefepime, FOX: cefoxitin, TZP: piperacillin-tazobactam, SXT: trimethoprim-sulfamethoxazole, MEM: meropenem, IMP: imipenem, EPT: ertapenem, CN: gentamicin, LEV: levofloxacin, AK: amikacin, ATM: aztreonam, TGC: tigecycline, CO: colisin FOS: fosfomycin; EC: *E*.*coli*

### Characterization of resistance genes, OMPs and efflux analysis

All isolates resistant to ertapenem (MICs > 8 mg/L) were positive in EDTA synergistic test, and seven of them were positive or weakly positive in the modified Hodge test (MHT). We confirmed the presence and production of metallo-β-lactamas (MBL) by sequencing. All 21 isolates carried *bla*NDM-1, and one isolate from cardiac surgery also carried *bla*IMP-4. In addition to MBL, we examined other types of β-lactamase, including ESBL and AMPC. The distributions of resistance genes in these strains are listed in Table 1. We identified the ESBL genes as *bla*CTX-M-15, *bla*TEM-1, and *bla*CTX-M-14 in 18 (85.7%), 19 (90.5%) and 3 (14.3%) isolates, respectively. Genotyping results of AMPC genes confirmed *bla*DHA-1 in 18 (85.7%) isolates. Moreover, 19 strains carried class 1 integron. Sequence data from the variable region showed the class 1 integron of all 17 isolates belonging to ST20 had no resistant gene cassettes. Only isolate H21, belonging to ST 17, carried two different gene cassettes (dfrA1 gene cassette encoding resistance determinants to trimethoprim and orfC gene cassette).

**Fig 1:**
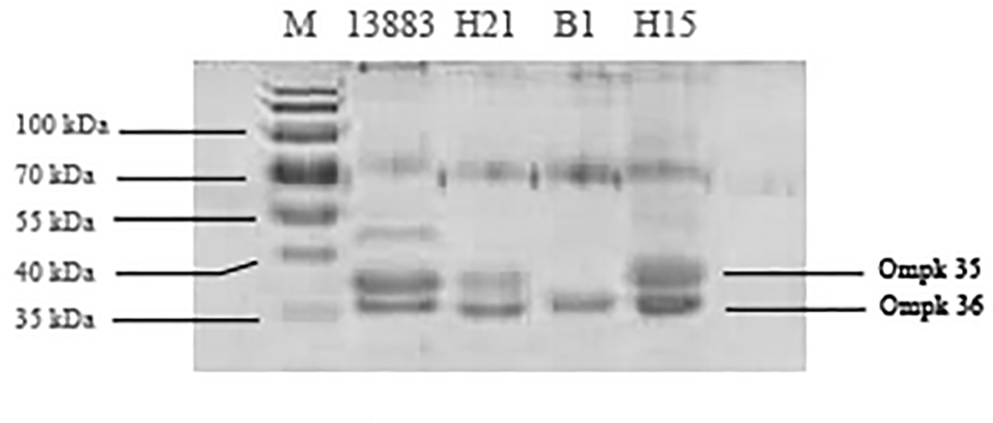
SDS-PAGE of outer membrane proteins. M: Marker; 13883: ATCC13883; B1: strain with OmpK36 deficiency.

We detected the genes of outer membrane proteins OmpK35 and OmpK36 in all 21 isolates. The base deletions of OmpK36 genes were observed in H21 and H15. OMPs of H21 and H15 were detected by SDS-PAGE. Compared with *K*. *pneumoniae* ATCC13883, OmpK35 and OmpK36 remained in H21 and H15 with molecular weights of 35–40 kDa (Fig. 1). We found no change in MIC for imipenem or meropenem in the absence or presence of CCCP.

### Association among sequence typing, resistance genes expression and antibiotic susceptibility

All 21 *K*. *pneumoniae* with *bla*NDM-1 were susceptible to levofloxacin, amikacin, tigecycline, and colisin. Among them, 17 isolates of ST20 carried *bla*CTX-M-15, *bla*DHA-1 and class 1 integron; and 15 isolates of ST20 also carried *bla*TEM-1. 17 isolates of ST20 were also susceptible to gentamicin and fosfomycin. Only isolate H1 was resistant to trimethoprim-sulfamethoxazole. Three *K*. *pneumoniae* of ST17 carried *bla*TEM-1 and *bla*CTX-M-14 in addition to *bla*NDM-1. Isolate H21 also carried *bla*IMP-4 and class 1 integron containing gene cassettes. Compared with ST20, *K*. *pneumoniae* ST17 did not carry *bla*DHA-1; and all ST17 were resistant to gentamicin and trimethoprim-sulfamethoxazole. Although one *K*. *pneumoniae* of ST54 produced the same β-lactamase gene as ST20, it was resistant to trimethoprim-sulfamethoxazole and fosfomycin. It is interesting to note that only *K*. *pneumoniae* ST54 was sensitive to aztreonam.

### Transfer of carbapenem resistance

Our results show that β-lactam resistance was successfully transferred to *E*. *coli* J53 from *K*. *pneumonia* in 20 isolates. Compared to the recipient strain *E*.*coli* J53, all *E*. *coli* transconjugants exhibited significantly reduced carbapenem susceptibility. MIC of imipenem, meropenem and etarpenem increased 8–64 fold, 32–512 fold and 256–2048 fold, respectively (Table 3). The sensitivities of cefotaxime, ceftazidime, cefepime, cefoxitin, piperacillin-tazobactam and aztreonam were similar to donor strains. All *E*. *coli* transconjugants showed positive EDTA synergistic tests with the same carbapenemase genes as the donor. Plasmid profiling revealed that transconjugants acquired single high-molecular-weight plasmids of approximately 336 kb.

**Table 3:** The results of antibiotic susceptibility testing of *E*.*coli* J53 transconjugant strains derived from blaNDM-producing *k*. *pneumonia* transconjugants (μg/ml).

isolate No.	IMP	MEM	ETP	CN	AK	TZP	CTX	CAZ	FEP	FOX	LEV	SXT	TGC	CO	FOS	ATM
H1-J53	2	2	>32	<1	<2	64	>256	>256	8	>256	<0.25	<0.5	0.25	0.5	2	32
H2-J53	2	2	>32	<1	<2	64	>256	>256	>256	>256	<0.25	<0.5	0.25	0.5	2	>256
H3-J53	2	4	4	<1	<2	64	>256	>256	8	>256	<0.25	<0.5	0.25	0.5	2	>256
H4-J53	>32	>32	>32	<1	<2	>256	>256	>256	>256	>256	<0.25	<0.5	0.25	0.5	2	>256
H5-J53	>32	>32	>32	<1	<2	>256	>256	>256	>256	>256	<0.25	<0.5	0.25	0.5	2	>256
H6-J53	2	2	16	<1	<2	>256	>256	>256	8	>256	<0.25	<0.5	0.25	0.5	2	>256
H7-J53	2	2	4	<1	<2	>256	>256	>256	>256	>256	<0.25	<0.5	0.25	0.5	2	>256
H8-J53	4	4	4	<1	<2	>256	>256	>256	>256	>256	<0.25	<0.5	0.25	0.5	2	>256
H9-J53	32	32	32	<1	<2	>256	>256	>256	>256	>256	<0.25	<0.5	0.25	0.5	2	>256
H10-J53	2	4	>32	<1	<2	>256	>256	>256	8	>256	<0.25	<0.5	0.25	0.5	2	>256
H11-J53	>32	>33	>33	<1	<2	>256	>256	>256	>256	>256	<0.25	<0.5	0.25	0.5	2	>256
H12-J53	>32	>32	>32	<1	<2	>256	>256	>256	>256	>256	<0.25	<0.5	0.25	0.5	2	>256
H13-J53	4	32	32	<1	<2	>256	>256	>256	>256	>256	<0.25	<0.5	0.25	0.5	2	1
H14-J53	32	32	>32	<1	<2	>256	>256	>256	>256	>256	<0.25	<0.5	0.25	0.5	2	>256
H15-J53	>32	>32	>32	<1	<2	>256	>256	>256	>256	>256	<0.25	<0.5	0.25	0.5	2	>256
H16-J53	>32	>32	>32	<1	<2	>256	>256	>256	>256	>256	<0.25	<0.5	0.25	0.5	2	>256
J17-J53	2	>32	>32	<1	<2	>256	>256	>256	32	>256	<0.25	<0.5	0.25	0.5	2	>256
H18-J53	>32	8	>32	<1	<2	>256	>256	>256	>256	>256	<0.25	<0.5	0.25	0.5	2	>256
H19-J53	>32	>32	>32	<1	<2	>256	>256	>256	>256	>256	<0.25	<0.5	0.25	0.5	2	>256
H20-J53	>32	4	32	<1	<2	>256	>256	>256	>256	>256	<0.25	<0.5	0.25	0.5	2	>256
EC J53	<1	<1	<0.5	<1	<2	<0.5	<1	<1	<1	<1	<0.25	<0.5	0.25	0.5	2	<1

MIC: Minimum inhibitory concentration, CTX: cefotaxime, CAZ: ceftazidime, FEP: cefepime, FOX: cefoxitin, TZP: piperacillin-tazobactam, SXT: trimethoprim-sulfamethoxazole, MEM: meropenem, IMP: imipenem, EPT: ertapenem, CN: gentamicin, LEV: levofloxacin, AK: amikacin, ATM: aztreonam, TGC: tigecycline, CO: colisin FOS: fosfomycin; EC J53: recipient

### Molecular epidemiology of 21 *K*. *pneumoniae*

**Fig 2:**
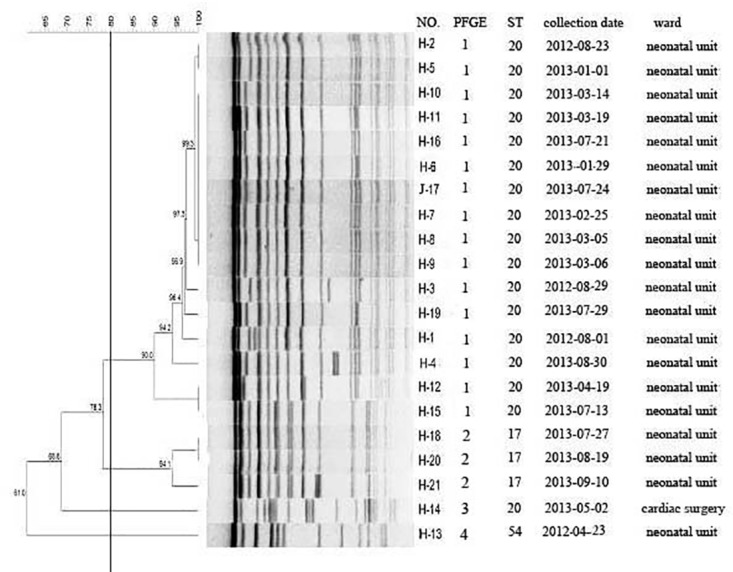
Dendrogram analysis. Dendrogram generated with the Fingerprinting II Informatix software package (Bio-Rad Laboratories, Hercules, CA) showing the relatedness of fingerprints (XbaI-PFGE) for 21 *K*. *pneumoniae*. The phylogenetic tree was constructed using the Dice coefficient and UPGMA clustering. A genetic similarity index scale is shown in the left of the dendrogram. PFGE types, strain number, collection data and wards distribution information are included along each PFGE lane. No., number

PFGE revealed 4 distinct clusters among the 21 *K*. *pneumoniae* isolates (Fig. 2). Strain H13, isolated from umbilicus, belonged to ST54, which was a different clone from the strains isolated from sputum and blood. Isolates H18, H20 and H21 belonged to the same clone, sharing the same sequence type as ST17. Isolate H21 was from cardiac surgery. Compared with PFGE that divided other strains into 2 different clusters, MLST assigned them into the same ST20 cluster. Our results show two clones of *K*. *pneumoniae* spread in the neonatal unit. Two outbreaks caused by *K*. *pneumoniae* (ST20) were noted, the first during August 2012 involving four patients and the second beginning in January 2013 involving twelve patients. During the second outbreak involving three patients (two from neonatal unit and one from cardiac surgery), the clone *K*. *pneumoniae* (ST17) was isolated. *K*. *pneumoniae* ST17 and ST20 caused newborn pneumonia. ST17 spread from the neonatal unit to the cardiac surgery unit. Fortunately, *K*. *pneumoniae* ST54, cause of neonatal umbilical infection, did not disseminate. Ultimately, only one patient died and all other patients were treated and discharged. Results show the source strain (J17) *K*. *pneumoniae*, belonging to ST20, was from the bed railing of patient (H16).

## Discussion

Carbapenem antibiotics are the primary treatment of serious infections caused by ESBL-producing gram-negative bacteria. Once carbapenem-resistant strains emerge, no drugs are available for treatment, with limited therapeutic choices including aminglycosides, polymxins, tigecycline, fosfomycin and temocillin. Therefore, the issue of carbapenem—resistance in enterobacteriaceae deserves special attention. In our hospital, *K*. *pneumoniae* producing *bla*NDM-1 was mainly derived from neonatal wards. The prematurity, very low body weight and poor immunity of newborns greatly increased chances of infection. The investigation of the surrounding environment revealed that the source of the outbreak was the bed railing of a patient who carried carbapenem-resistant *K*. *pneumoniae*, underscoring the importance of strict disinfection.

The use of antimicrobial drugs is a risk factor for infection of carbapenem—resistant enterobacteriaceae (CRE). Clinical data show that two weeks before patients were treated with cephalosporins, six patients used carbapenem antibiotics. Currently, no effective antimicrobials are available to treat CRE infection in newborns. CRE infections have a high mortality rate, approximately 70% in patients with bacteremia [18]. In this study, one patient with bloodstream infection died.

In our study, homology analysis indicated that CRE easily spread to different patients in the same ward, with a tendency to spread among multiple wards. Epidemic clones in this study were *K*. *pneumoniae* ST20 and ST17. Both have been previously identified among newborns affected by ESBL-*K*. *pneumoniae*, which produced *bla*CTXM-15 in Spain and Canada [19,20]. Here, we provide the first report confirming *bla*NDM-1-producing *K*. *pneumoniae* ST20 and ST17 caused an infection outbreak. Comparing ST20 and ST17, only the infB allele was different, with a base variant at 279 (T-C). Dortet L found that *Acinetobacter sp*. plays a key role in spreading *bla*NDM genes into enterobacteriaceae [21]. Our conjugation data indicates *bla*NDM-1 was transferred on a 336kb plasmid. Furthermore, two MBLs, *bla*IMP-4 and *bla*NDM-1, were initially co-expressed in a single carbapenem-resistant pathogen (*K*. *pneumoniae* ST17). However, the isolate carrying two MBLs and class 1 integron containing gene cassettes failed to transfer. We also found 3 strains of *K*. *pneumoniae* ST17 were resistant to gentamicin, and one strain was resistant to fosfomycin. Several gene cassettes encoding extended-spectrum beta-lactamases or carbapenemases have been described [22]. Most isolates with the class 1 integron in this study had no resistance gene cassettes. The spread of resistance genes was not due to class 1 integron.

Previous studies suggest OmpK plays an important role in the resistance or reduced susceptibility to carbapenems in *K*. *pneumoniae* isolates producing AmpC, ESBL or broad-spectrum *β*-lactamase [23, 24]. OmpK deficiency also contributes to the high-level carbapenem resistance in K. pneumoniae carrying blaKPC or blaIMP[25, 26]. In this study, although there was a high frequency of ESBL, AMPC and *bla*NDM-1 in 21 *K*. *pneumonia* isolates with high carbapenems MICs, we did not find a lack of outer-membrane proteins. We show base deletions of *bla*OmpK36 in H21 and H15, but we found no loss of protein. Shi et al suggests mutation or base deletions of protein-coding genes lead to alterations in the open reading frame, amino acid sequences and protein configuration [27]. Thus, the pore size of protein OmpK35 or OmpK36 can be affected and the accessibility of drugs can be impeded. In this study, no MIC changes of imipenem or meropenem were observed when CCCP was added. The results indicate that the resistance to carbapenems seems to be unrelated to efflux.

In conclusion, the production of *bla*NDM-1 coupled with *bla*DHA-1,*bla*CTX-M and *bla*TEM-1 β-lactamase genes plays an important role in conferring resistance of *K*. *pneumoniae* strains to carbapenems. The outbreak caused by *bla*NDM-1-producing *K*. *pneumoniae* highlights an urgent need to develop effective strategies for the prevention and control of infections. Our results show that strict disinfection of the environment and hand hygiene may be the most effective strategy in preventing CRE spreading. Limited application of antimicrobials, especially for carbapenems and cephalosporins, may reduce the appearance of CRE.
